# Altered Epigenetic Profiles in the Placenta of Preeclamptic and Intrauterine Growth Restriction Patients

**DOI:** 10.3390/cells12081130

**Published:** 2023-04-11

**Authors:** Carter Norton, Derek Clarke, Joshua Holmstrom, Isaac Stirland, Paul R. Reynolds, Tim G. Jenkins, Juan A. Arroyo

**Affiliations:** Department of Cell Biology and Physiology, Brigham Young University, Provo, UT 84602, USA

**Keywords:** placenta, epigenetics, pre-eclampsia, intrauterine growth restriction, DNA methylation

## Abstract

Intrauterine growth restriction (IUGR) and preeclampsia (PE) are placental pathologies known to complicate pregnancy and cause neonatal disorders. To date, there is a limited number of studies on the genetic similarity of these conditions. DNA methylation is a heritable epigenetic process that can regulate placental development. Our objective was to identify methylation patterns in placental DNA from normal, PE and IUGR-affected pregnancies. DNA was extracted, and bisulfite was converted, prior to being hybridized for the methylation array. Methylation data were SWAN normalized and differently methylated regions were identified using applications within the USEQ program. UCSC’s Genome browser and Stanford’s GREAT analysis were used to identify gene promoters. The commonality among affected genes was confirmed by Western blot. We observed nine significantly hypomethylated regions, two being significantly hypomethylated for both PE and IGUR. Western blot confirmed differential protein expression of commonly regulated genes. We conclude that despite the uniqueness of methylation profiles for PE and IUGR, the similarity of some methylation alterations in pathologies could explain the clinical similarities observed with these obstetric complications. These results also provide insight into the genetic similarity between PE and IUGR and suggest possible gene candidates plausibly involved in the onset of both conditions.

## 1. Introduction

Placental insufficiency is implicated in multiple pregnancy complications including preeclampsia (PE), intrauterine growth restriction (IUGR) and others [1]. PE occurs in up to 8% of total pregnancies and is characterized by high blood pressure (140 mm Hg systolic or 90 mm Hg diastolic) and the excretion of protein in the urine (>300 mg in 24 h) after the 20th week of pregnancy [2,3,4]. IUGR affects up to 12% of all pregnancies and is characterized by low fetal growth (<10th percentile) and increased risk of fetal and neonatal morbidity and mortality [5,6]. Additionally, several studies have reported long-term sequela of IUGR complications including adult hypertension, heart disease, stroke, and diabetes [7,8,9,10,11]. While IUGR and PE are different obstetric complications, they exhibit similar pathologies including placental apoptosis, increased placental inflammation, and abnormal vascularization of maternal placenta tissues [6,7,8]. Vascularization of the placenta is a process that relies on placental trophoblast invasion into uterine tissues. These trophoblasts, following invasion, facilitate the remodeling of the spiral artery networks within the placenta. Vascular remodeling leads to increased blood flow in the placenta, maternal blood pressure, and nutrient delivery to the growing fetus [9]. In cases of PE and IUGR, this vascularization process is upregulated which leads to hypertensive gestational symptoms. Since PE and IUGR are present in patients with abnormal vascularization of placental tissues, it is plausible that both pathologies could result from similar instigating factors.

Alterations to the epigenetic profile of placental tissues have been reported in cases of PE and IUGR [10,11,12]. The presence of epigenetic abnormalities in both PE and IUGR proposes a possible correlational relationship between epigenomic alterations and PE/IUGR pathologies. DNA methylation is an epigenetic modification that affects genome architecture and can influence gene transcription [13]. This process occurs on the cytosine residues of CG dinucleotides and, in part, regulates gene expression from DNA [14]. The DNA methylation profile in placental tissues changes throughout gestation. These changes are mostly attributed to the fact that gene expression is different amongst the various placental cell types and the cellular composition of the placenta changes throughout gestation [13,15]. Previous methylation studies of PE and IUGR in human placental cells show that methylation can be a marker of intrauterine health and plays a critical functional role in fetal development [16,17,18]. Our objective was to compare the methylation profiles of human placental tissues from PE, IUGR and control pregnancy groups. Most importantly, we are assessing both IUGR and PE in parallel to identify similarities in aberrant DNA methylation. Considering the combination of the Illumina Methylation EPIC array, GREAT analysis, and our parallel assessment of both IUGR and PE, this study reaches a depth and breadth of epigenomic analysis not previously reported.

## 2. Materials and Methods

### 2.1. Human Placental Tissues

All frozen human placental samples (PE, IUGR, and control) were purchased from the Research Center for Women’s and Infant’s Health BioBank, Ontario, Canada. We analyzed six samples from each cohort (control, PE, and IUGR). A total of 18 samples were taken through the statistical analysis outlined below. Samples were collected from placentas delivered in conjunction with the delivery of the fetus either vaginally or by C-section. PE diagnosis was based on elevated blood pressure (systolic blood pressure > 160 mm Hg and/or a diastolic blood pressure > 110 mmHg) and proteinuria (≥5). IUGR placentas were confirmed by ultrasound showing placental insufficiency with uterine Doppler and absent end diastolic flow (AEDV) and an estimated fetal weight below the 10th percentile. Sample demographics and clinical data are shown in Table 1.

### 2.2. DNA Extraction

Placental tissues were incubated with proteinase K (Qiagen) at 56 °C overnight. DNA was then extracted using the DNeasy Blood and Tissue kit using the manufacturer’s recommended protocol for simple column-based DNA isolation. Genomic DNA was extracted from lysed placental tissue using the Qiagen DNA isolation.

### 2.3. Bisulfite Conversion

A total of 500 ng of extracted DNA was bisulfite converted using the EZ-96 DNA Methylation kit Gold Kit (Zymo Research) according to the manufacturer’s recommendations. Bisulfite-converted DNA was then hybridized to Illumina’s Epic 850K Methylation array microarrays (Illumina, San Diego, CA, USA). Array hybridization and analysis were performed according to Illumina protocols at the University of Utah Genomics Core Facility.

### 2.4. Data Processing and Global Epigenetic Analyses

Following array processing, raw intensity values for methylation at each CpG site were assessed using the minfi package in R 4.1.2 [19,20]. Intensity values were used to generate fraction methylation values (β-values). This process utilized SWAN normalization to ensure that datasets were comparable. The generated β-values range from 0 to 1, with 0 indicating a completely unmethylated CpG site and 1 indicating a completely methylated CpG. The resulting β-values were used to perform various epigenetic analyses at global, regional, and gene-specific levels. Global methylation analysis was performed in each group to determine if PE or IUGR were correlated with changes in overall methylation, and methylation at specific genomic features (CpG islands, gene body, promoters, etc.) as compared to controls.

### 2.5. USEQ Sliding Window Analysis

A sliding window analysis was used to identify regional methylation changes between the control group and both PE and IUGR. This analysis is performed through the USeq bioinformatics software package with both the Methylation Array Scanner and the Enriched Region Maker applications [21]. In brief, this software utilizes a sliding window approach to identify the boundaries of regional differential methylation in the genome between two groups. The regional differential methylation (both hypomethylation and hypermethylation) was identified based on a Wilcoxon signed rank analysis, with significance based on the following thresholds: Phred-scaled FDR of ≥13 which correlates with a *p*-value of ~0.05, absolute log2 ratio of ≥0.2 (representing a 15% change in methylation level), and a total number of CpGs in any significant window of ≥3. Significant regions were displayed in a genome-wide Circos plot, produced with the BioCircos package in R 4.1.2 [22].

### 2.6. Great Analysis

A Stanford GREAT analysis [23] was performed using each of the significant differentially methylated regions, in order to predict possible biological functions connected with non-coding regions of those regions (“cis-regulatory regions”).

### 2.7. Immunoblot

Western blot analysis was used to determine the expression level of FAN1 in control, IUGR and PE placental samples, NAPRT1 and HIST1H4L in Control and PE samples and CRABP1 in IUGR and control samples as previously described (n = 10) [1]. Cell lysates (50 μg) were separated on a 10% SDS-Page gel and transferred onto nitrocellulose membranes. Membranes were blocked and incubated overnight with antibodies against FAN, NAPRT1, HIST1H4L, CRABP1 (Abcam Waltham, MA), or β-actin (Santa Cruz Biotechnology, Dallas, Texas). Membranes were then incubated with secondary IRDye antibodies (680RD donkey-anti goat and 680RD donkey-anti rabbit; LICOR Lincoln, NE) at room temperature for an hour. Membranes were developed on a Li−COR Odyssey CLx. All results were normalized to β-actin as our loading control. Fluorescence density comparisons were made between the treated and control groups.

### 2.8. Statistical Analysis for Immunoblot

Data are shown as means ± SE. Differences in CRABP1, NAPRT1, HIST1H4L, and FAN1 expression were determined between control and disease placenta using Mann–Whitney tests. Significant differences between groups were noted at *p* < 0.05. Statistical analysis was performed with GraphPad Prism 7.0

## 3. Results

### 3.1. Global Methylation Analysis

**Figure 1:**
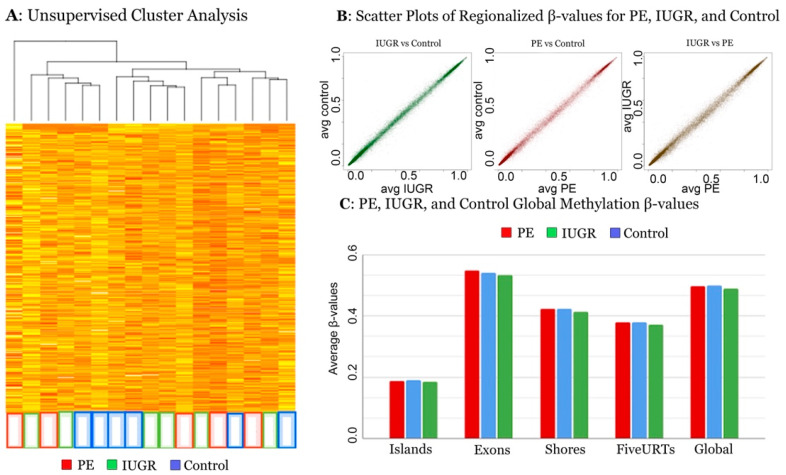
Comparative analyses of global profiles for PE, IUGR, and control placental tissues. Unsupervised cluster analysis of regionalized β-values for samples with a dendrogram indicating similarity (**A**). Scatter plots of regionalized β-values for IUGR vs Control, PE vs Control, and IUGR vs PE, respectively (**B**). Bar plots of average β-values at five global regions of interest (**C**).

An unsupervised cluster analysis based on methylation values was performed on all samples, generating a clustered heatmap using R’s *heatmap* function. Except for a single cluster of four control samples, no clustering of samples was observed (Figure 1A). Next, we performed a comparison of regionalized β-values between IUGR, PE, and control groups in the context of large genomic and CpG island associated features. This comparison revealed slightly more variation at intermediately methylated regions compared to hyper and hypomethylated regions but no significant deviation from expected correlations between the various groups (Figure 1B). Regionalized β-values were observed to vary slightly more between IUGR and PE than between IUGR and control or PE and IUGR (Figure 1B).

To better clarify our results, we analyzed the methylation of each sample group and compared globally and at specific genomic features, including gene bodies, CpG islands, CpG shores, and five prime untranslated regions (5UTRs). Average global β-values at genomic features are shown in Figure 1C. No significant association was found in either global methylation or in any specific genomic features in samples for all groups (Figure 1C). Each sample group was differentially methylated between genomic features. Average β-values were lowest for all samples at CpG islands, and the most methylated at gene bodies (Figure 1C).

### 3.2. Regional Methylation Analysis

**Figure 2:**
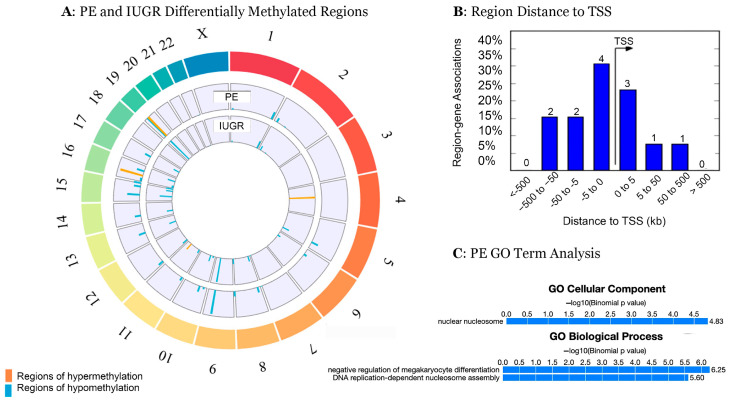
Comparative methylation profiles and ontological analysis of placental tissues. Circos Plot indicating regions of significant hyper and hypomethylation (FDR > 13) across the genome in PE (outer ring) and IUGR (inner ring) (**A**). Height of region indicates the size of variance from control (log2ratio). Distance of each region from transcription start site of the most differentially methylated regions (FDR > 40) (**B**). GO term analysis of the most differentially methylated regions for PE (**C**).

We next determined differentially methylated regions between our groups. USEQ’s Methylation Array Scanner and Enriched Region Maker applications identified 81 differentially methylated regions between PE, IUGR, and control groups, including hypo and hyper-methylated regions (Figure 2A). Regions are displayed on their respective genome locations in the Circos plot, with regions associated with IUGR on the inner track and PE on the outer track. Hypomethylated regions are marked blue, hypermethylated are marked orange, and respective log2 ratio values are indicated by region height (maximum value of 1.77; Figure 2A). Fifty-one differentially methylated regions were found between PE and control, and 30 differentially methylated regions were found between IUGR and control. Five of the regions of interest are hypomethylated for both IUGR and PE. Nine hypomethylated regions had Phred-scaled FDR scores ≥ 40 (equivalent to a *p*-value of ~0.0001) and were selected for further analysis (Table 2). Hypomethylated regions were found to be mostly within 500 bases of a transcription start site (TSS) which is often, though not always, associated with increased gene expression (Figure 2B). Subsequent GREAT analysis was performed for IUGR and PE groups using each of the nine most hypomethylated regions. The GREAT analysis did not find any gene ontology terms or cellular pathways that correlated with the IUGR in hypomethylated regions (not shown). There were also no gene ontology terms or cellular pathways identified for shared high FDR regions. For the PE samples, the GREAT analysis indicated that the regions selected were related to nuclear nucleosome regulation, as well as the negative regulation of megakaryocyte differentiation, and DNA replication-dependent nucleosome assembly (Figure 2C).

**Figure 3:**
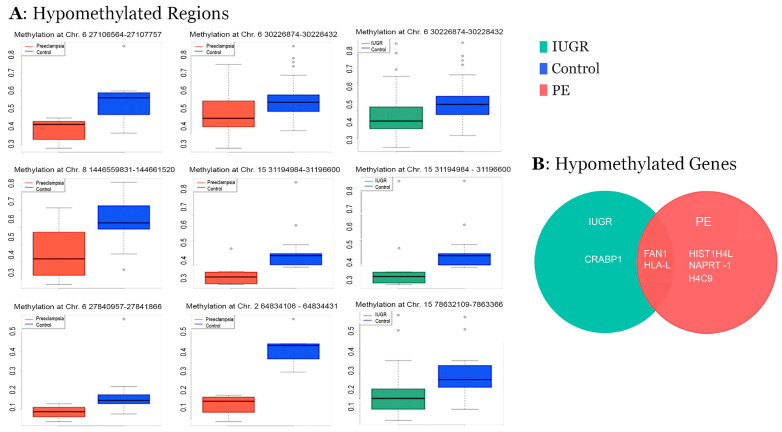
Shared areas of hypomethylation at key regulatory regions in both PE and IUGR placental tissues. Bar plots indicating methylation beta-values at significantly hypomethylated regions (FDR > 40) in IUGR and PE samples compared with control (**A**). Venn diagram of each hypomethylated gene promoter region associated with PE and IUGR (**B**).

**Figure 4:**
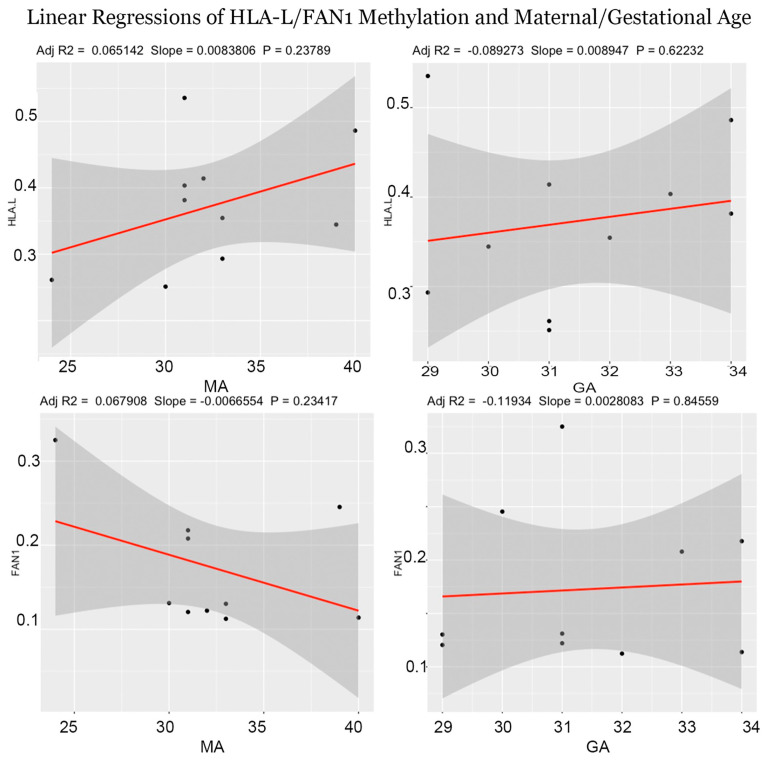
Linear regressions comparing FAN1/HLA-L gene promoter hypomethylation to maternal age (MA)/gestational age (GA). Plot legends include adjusted R^2^ values, slopes, and *p*-values.

Box plots were used to represent mean beta values at differentially methylated regions. We generated box plots for average methylation values for all CpG sites in each region. Regions associated with PE are shown in red, IUGR in green, and control in blue. These plots depict statistically significant differences between the control group and either the PE or IUGR group. (Figure 3A). Of those regions, one IUGR region was associated with the promoter regions of the CRABP1 gene (Figure 3B). Three of the six PE regions were associated with either HIST1H4L, NAPRT1, or H4C9 gene promoter (Figure 3B). One hypomethylated region in the PE was not associated with a gene-annotated region. Two hypomethylated regions shared between IUGR and PE groups were associated with a single gene promoter region each (FAN1 and HLA-L) (Figure 3B). A linear regression model and an adjusted correlation (adjusted R^2) coefficient were generated for the two shared genes FAN1 and HLA-L, and maternal age (MA) and gestational age (GA) of each IUGR and PE sample (Figure 4). Control samples were excluded because they had no variation in gestational age (38 weeks), and limited variation in maternal age. No significant correlation was found between either gene and GA or MA (minimum *p*-value: 0.234) (Figure 4). In addition, the maximum adjusted correlation coefficient was 0.067 for FAN1 and maternal age (Figure 4). Linear regressions were generated using the ggplot2 package (_26_) in R 4.1.2.

### 3.3. Immunoblot for CRABP1, FAN1, HISTH4L and NAPRT1

**Figure 5:**
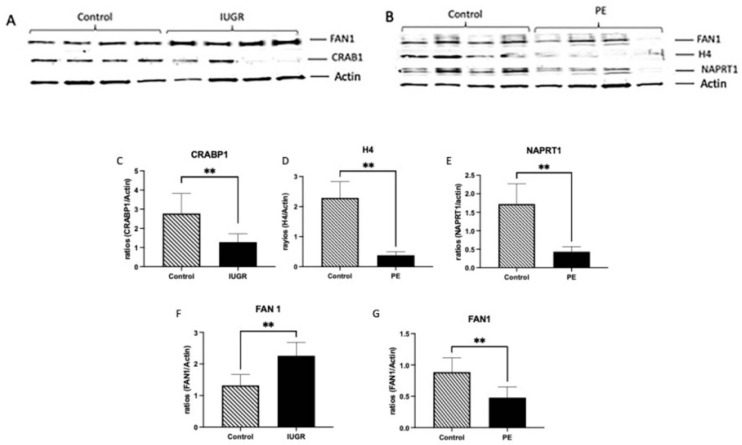
FAN1, CRABP1, HIST1H4L and NAPRT1 proteins in IUR, PE and control placentas. A representative western blot for these proteins is shown in (**A**,**B**). CRABP1 protein was decreased in the IUGR placenta (**C**). HIST1H4L protein was decrease while NAPRT1 protein was increased in the PE placenta (**D**,**E**). FAN1 protein was increased in IUGR and decreased in PE Placenta (**F**,**G**). Representative data are shown with ** *p* ≤ 0.05.

Western blot was performed to determine the protein expression of genes with methylation in IUGR (CRABP1), PE (HIST1H4L and NAPRT1) and a commonly shared gene (FAN1). A characteristic Western bot is shown in Figure 5A,B. The CRABP1 gene produces the Cellular retinoic acid-binding protein 1. This protein is responsible for the delivery of Retinoic Acid (RA) to its receptors thereby regulating its bioavailability and metabolism [24,25]. RA is important for the development of a healthy placenta by regulating the function of trophoblast placental cells [26]. We observed that CRABP1 protein was decreased (2.2-fold; *p* < 0.004) in the IUGR placenta compared to controls (Figure 5C). HIST1H4L encodes the Histone 4 (H4) protein. H4 is one of the Histone core proteins that form the nucleosome [27]. Depletion of H4 is known to induce genome instability by increasing homologous recombination [27]. We observed a 6.0-fold H4 decrease (*p* < 0.02) in the PE placenta as compared to the control (Figure 5D). The NAPRT1 gene encodes the Nicotinate Phosphoribosyltransferase protein (NAPRT). This protein is associated with the prevention of oxidative stress [28]. In the PE placenta, there was a decrease (4-fold; *p* < 0.003) in NAPRT1 compared to controls (Figure 5E). FAN1 encodes the FANCD2/FANCI-associated nuclease 1 enzyme (FAN1). Mutations in FAN1 are associated with the development of disease due to impaired DNA damage repair [29,30,31]. In the IUGR placenta, FAN1 protein was increased (1.7-fold; *p* < 0.002) compared to controls (Figure 5F). In contrast, FAN1 protein was decreased (1.8-fold; *p* < 0.002) in the PE placenta compared to controls (Figure 5G)

## 4. Discussion

This study combined epigenetic analyses and protein expression assays to examine the relationship between epigenetic dysregulation in PE and IUGR conditions. Due to the role of methylation in gene expression throughout the gestational process and as a potential instigator of PE and IUGR pathologies, an epigenetic analysis could be very important for understanding placental development, assessing placental health, and providing clues about intrauterine exposures [15]. Many previous DNA methylome studies have used the Illumina Infinium HumanMethylation450 BeadChip to carry out methylation analysis; however, this study used the newer Illumina Infinium Methylation EPIC array which analyzes over 850,000 CpG sites, nearly twice the amount of the 450K BeadChip. This more thorough analysis can reach regions more distant from the promoter than the 450 K analysis [32]. Additionally, we used the GREAT analysis from Stanford University, which considers distal binding sites, annotations from 20 ontologies, and false positives, each of which are features not available in other packages [23]. Initial methylation analyses identified six DMRs in PE and three DMRs in IUGR placental tissues compared to the control. Eight of these regions were found to be located on gene promoters or exons, suggesting the potential for some influence on gene expression. Only two of these eight gene-related regions were common to both PE and IUGR pathologies. Regions on two gene promoters (FAN-1 and HLA-L pseudogene) were hypomethylated for both PE and IUGR. A GREAT analysis and ontological study of PE DMRs found a correlation between these regions and nucleosome assembly and structure.

While other research has identified differential gene methylation in PE and IUGR, epigenetic studies comparing both disease states have been limited to an analysis of individual CpG sites only [33,34,35,36]. Interestingly, prior research has not implicated methylation or expression of either FAN-1 or HLA-L in either PE or IUGR, suggesting the need for further research into the regulatory mechanisms for both diseases. To explore possible mechanistic explanations for the similarity in PE and IUGR symptomatology immunoblot assays were conducted on PE, IUGR, and control placental tissues. These assays confirmed the results attained through epigenetic analysis; that aberrant gene expression at specific loci is correlated with the onset of PE and IUGR conditions. In both PE and IUGR conditions, hypomethylation existed at all differentially methylated regions of significance. In many cases, hypomethylation at a promoter region corresponds with increased expression of that gene; however, the opposite trend occurred in some of our studied targets.

Immunoblot assays revealed an increase in FAN1 expression in IUGR tissues and a decrease in FAN1 expression in PE tissues. FAN1 is a conserved nuclease involved in DNA damage response and repair of both single-stranded (ssDNA) and double-stranded (dsDNA) DNA damage [37,38,39]. Mutations of the FAN1 gene lead to several diseases and organ damage [40]. Interestingly we observed an increase in this protein in IUGR tissues. This suggests that perhaps FAN1 could be upregulated as a mechanism of protection in the placenta during this disease. Currently, there are no real data on DNA methylation regulation specifically at FAN1 so the only thing we have to work off of is the standard mechanism for methylation regulation, specifically that gene promoter DNA methylation loss results in increased gene expression. While this is commonly true, this is not always the case. In some cases, genes are not highly regulated by DNA methylation or may be more influenced by other mechanisms (histone modifications or transcription factors). In addition, it is possible that even if regulated by methylation, the initial wave of methylation-regulated gene transcription has already been completed and the current state of the cell does not necessarily reflect this activation or repression. As a result, it is difficult to know what specifically is driving the alterations in protein levels that we have seen with FAN1 in the IUGR placenta and how this also does not comport directly with the patterns we would typically expect to see in the regulation of gene expression by DNA methylation. Previous studies have shown increased placental DNA damage including increases in dsDNA damage in the PE placenta [41,42]. The fact that FAN1 expression is decreased suggests that perhaps this could be a factor involved in the increased placental DNA damage during this disease. The combined results of protein assays and epigenetic analyses support the idea that FAN1 plays a regulatory role in the onset of both disease states. This is novel as FAN1 protein correlation with placental disease has not been previously shown and merits more investigation.

Our data clearly indicate that hypomethylation at several gene promoters in placental DNA is associated with IUGR and PE, suggesting that the promoters may be indicative of disease progression for both diseases. Few genes were uniquely hypomethylated in IUGR or PE placentas. CRAB1 was a gene hypomethylated only in the IUGR placenta. As previously mentioned, this gene produces the cellular retinoic acid-binding protein that is responsible for the delivery of Retinoic Acid (RA) to its receptors [42]. In our studies, hypomethylation of this gene led to decreased expression of the CRAB1 protein. Although there is not much research about this protein and IUGR, studies have reported a decrease in RA signaling responses in humans with IUGR [27]. In the PE placenta, we observed unique hypomethylation of HISTH4L, NAPRT1, and H4C9. As previously mentioned, the HISTH4L gene encodes the H4 protein, while H4C9 encodes a gene that is part of the H4 family. H4 is part of the core histones that form the nucleosome involved in genome integrity [43,44]. Previous studies have demonstrated that mutations in H4 affect DNA damage responses and impair cell survival [45,46]. We observed decreases in the H4 protein in the PE placenta tissues. This is of interest as we have previously shown that PE placentas are associated with increased DNA damage [42]. Although there are no previous reports establishing a direct connection between H4 and PE, we can speculate that as observed with FAN1, decreased H4 could be associated with the increased DNA damage observed in PE placentas. Our studies also demonstrated PE placenta hypomethylation for NAPRT1 and this was associated with decreased NAPRT protein in these tissues. The observed NAPRT1 hypomethylation is consistent with previous placental studies comparing PE and controls tissue but no protein levels were reported in these studies [47]. NAPRT1 is a cellular metabolic enzyme expressed in the placental trophoblast, which is known to prevent oxidative stress in several diseases including cancer [29]. This is important because the PE placenta is known to have high oxidative stress during this disease [48,49]. The fact that the NAPRT1 protein was decreased could drive the idea that this protein may be involved in the increased oxidative stress leading to the increased reactive oxidative species observed in this disease. These novel results portray NAPRT1 as a possible avenue of study in the placenta during PE.

There are a few important limitations to this study. An important consideration is that the placenta samples were collected post-partum and are limited in temporal scope. Prior research indicates that methylation changes occur throughout pregnancy [34], and longitudinal studies that analyze methylation signatures of patients through gestation could be important in determining when and how methylation changes occur.

The potential applications of this study to human health and patient diagnostics are substantial. Past research has shown liquid biopsy as a possible diagnostic technique for PE [50,51,52]. These assays rely on cell-free nucleic acids in blood plasma to identify differentially methylated regions indicative of disease states. The regions identified in this study could provide “target” areas of hypomethylation for future liquid biopsies that could be used in the research of genes affected by this disease [53,54].

## Figures and Tables

**Table 1:** Demographical and clinical patient data from the collected placental samples.

	Control	IUGR	PE
Maternal Age	34 ± 2.8	30 ± 1.3	36 ± 1.7
Gestational Age (wks)	38 ± 0.01	31 ± 0.5	32 ± 1.02
Fetal Weight (g)	3498 ± 45	972 ± 40.1	1813 ± 301
BMI (gestational)	22 ± 0.9	28 ± 4.64	38 ± 3.6
Blood pressure (Average S/D)	122/83	127/79	161/107
Umbilical Artery Resistance (Average Doppler)	NA	1.7 ± 0.14	NA
Proteinuria	Trae	Trace	+3 to +4
Female/Male ratios	1.0	0.8	1.0

%C-section/Vaginal (for all samples)—85%/15%.

**Table 2:** Hypomethylated regions with FDR scores ≥ 40.

**PE**							
Chromosome	Num. Array Cpgs	Start Loc.	Stop Loc.	Associated Genes	Region	Log2 Ratio	Phred-FDR
chr15	14	31194984	31196600	FAN1	Promoter	1.605	44.51
chr6	15	27840957	27841866	HIST1H4L	Promoter	0.841	44.51
chr6	43	30226874	30228432	HLA-L	Promoter	0.562	44.51
chr2	5	64834106	64834431	None	NA	0.646	44.25
chr8	15	144659831	144661520	NAPRT-1	Promoter	1.285	44.06
chr6	11	27106564	27107757	H4C9	Promoter	.476	40.26
**IUGR**							
Chromosome	Num. Array Cpgs	Start Loc.	Stop Loc.	Associated Genes	Region	Log2 Ratio	Phred-FDR
chr15	14	31194984	31196600	FAN1	Promoter	1.644	43.05
chr15	17	78632109	78633663	CRABP1	Promoter	0.971	43.05
chr6	43	30226874	30228432	HLA-L	Promoter	0.538	43.05

## Data Availability

All data discussed are presented within the article.

## References

[1] Tsai K., Tullis B., Jensen T., Graff T., Reynolds P., Arroyo J. (2021). Differential expression of mTOR related molecules in the placenta from gestational diabetes mellitus (GDM), intrauterine growth restriction (IUGR) and preeclampsia patients. Reprod. Biol..

[2] Bahr B.L., Price M.D., Merrill D., Mejia C., Call L., Bearss D., Arroyo J. (2014). Different expression of placental pyruvate kinase in normal, preeclamptic and intrauterine growth restriction pregnancies. Placenta.

[3] Fox R., Kitt J., Leeson P., Aye C.Y.L., Lewandowski A.J. (2019). Preeclampsia: Risk Factors, Diagnosis, Management, and the Cardiovascular Impact on the Offspring. J. Clin. Med..

[4] Lee G.T., Price M.D., Mejia C.A., Galan H.L., Arroyo J.A. (2014). Increased trophoblast expression of NFAT5/TonEBP in pre-eclamptic placentas and hyperosmolar-treated BeWo cells. Eur. J. Obstet. Gynecol. Reprod. Biol..

[5] Tsai K.Y.F., Tullis B., Mejia J., Reynolds P.R., Arroyo J.A. (2021). Regulation of trophoblast cell invasion by Pyruvate Kinase isozyme M2 (PKM2). Placenta.

[6] Romo A., Carceller R., Tobajas J. (2009). Intrauterine growth retardation (IUGR): Epidemiology and etiology. Pediatr. Endocrinol. Rev..

[7] Krebs C., Macara L.M., Leiser R., Bowman A.W., Greer I.A., Kingdom J.C. (1996). Intrauterine growth restriction with absent end-diastolic flow velocity in the umbilical artery is associated with maldevelopment of the placental terminal villous tree. Am. J. Obstet. Gynecol..

[8] Phipps E., Prasanna D., Brima W., Jim B. (2016). Preeclampsia: Updates in Pathogenesis, Definitions, and Guidelines. Clin. J. Am. Soc. Nephrol..

[9] Kaufmann P., Black S., Huppertz B. (2003). Endovascular trophoblast invasion: Implications for the pathogenesis of intrauterine growth retardation and preeclampsia. Biol. Reprod..

[10] Lim J.H., Kang Y.J., Bak H.J., Kim M.S., Lee H.J., Kwak D.W., Han Y.J., Kim M.Y., Boo H., Kim S.Y. (2020). Epigenome-wide DNA methylation profiling of preeclamptic placenta according to severe features. Clin. Epigenet..

[11] Nelissen E.C., van Montfoort A.P., Dumoulin J.C., Evers J.L. (2011). Epigenetics and the placenta. Hum. Reprod. Update.

[12] Novielli C., Mando C., Tabano S., Anelli G.M., Fontana L., Antonazzo P., Miozzo M., Cetin I. (2017). Mitochondrial DNA content and methylation in fetal cord blood of pregnancies with placental insufficiency. Placenta.

[13] Bianco-Miotto T., Mayne B.T., Buckberry S., Breen J., Rodriguez Lopez C.M., Roberts C.T. (2016). Recent progress towards understanding the role of DNA methylation in human placental development. Reproduction.

[14] Koukoura O., Sifakis S., Spandidos D.A. (2012). DNA methylation in the human placenta and fetal growth (review). Mol. Med. Rep..

[15] Robinson W.P., Price E.M. (2015). The human placental methylome. Cold Spring Harb. Perspect. Med..

[16] Banister C.E., Koestler D.C., Maccani M.A., Padbury J.F., Houseman E.A., Marsit C.J. (2011). Infant growth restriction is associated with distinct patterns of DNA methylation in human placentas. Epigenetics.

[17] Chu T., Bunce K., Shaw P., Shridhar V., Althouse A., Hubel C., Peters D. (2014). Comprehensive analysis of preeclampsia-associated DNA methylation in the placenta. PLoS ONE.

[18] Anderson C.M., Ralph J.L., Wright M.L., Linggi B., Ohm J.E. (2014). DNA methylation as a biomarker for preeclampsia. Biol. Res. Nurs..

[19] Aryee M.J., Jaffe A.E., Corrada-Bravo H., Ladd-Acosta C., Feinberg A.P., Hansen K.D., Irizarry R.A. (2014). Minfi: A flexible and comprehensive Bioconductor package for the analysis of Infinium DNA methylation microarrays. Bioinformatics.

[20] Fortin J.P., Triche T.J., Hansen K.D. (2017). Preprocessing, normalization and integration of the Illumina HumanMethylationEPIC array with minfi. Bioinformatics.

[21] Nix D.A., Courdy S.J., Boucher K.M. (2008). Empirical methods for controlling false positives and estimating confidence in ChIP-Seq peaks. BMC Bioinform..

[22] Cui Y., Chen X., Luo H., Fan Z., Luo J., He S., Yue H., Zhang P., Chen R. (2016). BioCircos.js: An interactive Circos JavaScript library for biological data visualization on web applications. Bioinformatics.

[23] McLean C.Y., Bristor D., Hiller M., Clarke S.L., Schaar B.T., Lowe C.B., Wenger A.M., Bejerano G. (2010). GREAT improves functional interpretation of cis-regulatory regions. Nat. Biotechnol..

[24] Enikeev A.D., Komelkov A.V., Axelrod M.E., Galetsky S.A., Kuzmichev S.A., Tchevkina E.M. (2021). CRABP1 and CRABP2 Protein Levels Correlate with Each Other but Do Not Correlate with Sensitivity of Breast Cancer Cells to Retinoic Acid. Biochemistry.

[25] Lin Y.L., Lin Y.W., Nhieu J., Zhang X., Wei L.N. (2020). Sonic Hedgehog-Gli1 Signaling and Cellular Retinoic Acid Binding Protein 1 Gene Regulation in Motor Neuron Differentiation and Diseases. Int. J. Mol. Sci..

[26] Huebner H., Hartner A., Rascher W., Strick R.R., Kehl S., Heindl F., Wachter D.L., Beckmann Md M.W., Fahlbusch F.B., Ruebner M. (2018). Expression and Regulation of Retinoic Acid Receptor Responders in the Human Placenta. Reprod. Sci..

[27] Kumar K., Moirangthem R., Kaur R. (2020). Genome protection: Histone H4 and beyond. Curr. Genet..

[28] Duarte-Pereira S., Silva S.S., Azevedo L., Castro L., Amorim A., Silva R.M. (2014). NAMPT and NAPRT1: Novel polymorphisms and distribution of variants between normal tissues and tumor samples. Sci. Rep..

[29] Segui N., Mina L.B., Lazaro C., Sanz-Pamplona R., Pons T., Navarro M., Bellido F., Lopez-Doriga A., Valdes-Mas R., Pineda M. (2015). Germline Mutations in FAN1 Cause Hereditary Colorectal Cancer by Impairing DNA Repair. Gastroenterology.

[30] Zhou W., Otto E.A., Cluckey A., Airik R., Hurd T.W., Chaki M., Diaz K., Lach F.P., Bennett G.R., Gee H.Y. (2012). FAN1 mutations cause karyomegalic interstitial nephritis, linking chronic kidney failure to defective DNA damage repair. Nat. Genet..

[31] Wang R., Persky N.S., Yoo B., Ouerfelli O., Smogorzewska A., Elledge S.J., Pavletich N.P. (2014). DNA repair. Mechanism of DNA interstrand cross-link processing by repair nuclease FAN1. Science.

[32] Pidsley R., Zotenko E., Peters T.J., Lawrence M.G., Risbridger G.P., Molloy P., Van Djik S., Muhlhausler B., Stirzaker C., Clark S.J. (2016). Critical evaluation of the Illumina MethylationEPIC BeadChip microarray for whole-genome DNA methylation profiling. Genome Biol..

[33] Almomani S.N., Alsaleh A.A., Weeks R.J., Chatterjee A., Day R.C., Honda I., Homma H., Fukuzawa R., Slatter T.L., Hung N.A. (2021). Identification and validation of DNA methylation changes in pre-eclampsia. Placenta.

[34] Del Vecchio G., Li Q., Li W., Thamotharan S., Tosevska A., Morselli M., Sung K., Janzen C., Zhou X., Pellegrini M. (2021). Cell-free DNA Methylation and Transcriptomic Signature Prediction of Pregnancies with Adverse Outcomes. Epigenetics.

[35] Chen P.Y., Chu A., Liao W.W., Rubbi L., Janzen C., Hsu F.M., Thamotharan S., Ganguly A., Lam L., Montoya D. (2018). Prenatal Growth Patterns and Birthweight Are Associated With Differential DNA Methylation and Gene Expression of Cardiometabolic Risk Genes in Human Placentas: A Discovery-Based Approach. Reprod. Sci..

[36] van den Berg C.B., Herzog E.M., Duvekot J.J., van der Spek P.J., Steegers E.A.P., Stoop M.P., Willemsen S.P., Steegers-Theunissen R.P.M. (2020). Differences in DNA methylation of insulin-like growth factor 2 and cadherin 13 in patients with preeclampsia. Pregnancy Hypertens..

[37] Jin H., Cho Y. (2017). Structural and functional relationships of FAN1. DNA Repair.

[38] Airik R., Schueler M., Airik M., Cho J., Porath J.D., Mukherjee E., Sims-Lucas S., Hildebrandt F. (2016). A FANCD2/FANCI-Associated Nuclease 1-Knockout Model Develops Karyomegalic Interstitial Nephritis. J. Am. Soc. Nephrol..

[39] Deshmukh A.L., Porro A., Mohiuddin M., Lanni S., Panigrahi G.B., Caron M.C., Masson J.Y., Sartori A.A., Pearson C.E. (2021). FAN1, a DNA Repair Nuclease, as a Modifier of Repeat Expansion Disorders. J. Huntingt. Dis..

[40] Thongthip S., Bellani M., Gregg S.Q., Sridhar S., Conti B.A., Chen Y., Seidman M.M., Smogorzewska A. (2016). Fan1 deficiency results in DNA interstrand cross-link repair defects, enhanced tissue karyomegaly, and organ dysfunction. Genes Dev..

[41] Furness D.L., Dekker G.A., Roberts C.T. (2011). DNA damage and health in pregnancy. J. Reprod. Immunol..

[42] Tsai K.Y.F., Tullis B., Breithaupt K.L., Fowers R., Jones N., Grajeda S., Reynolds P.R., Arroyo J.A. (2021). A Role for RAGE in DNA Double Strand Breaks (DSBs) Detected in Pathological Placentas and Trophoblast Cells. Cells.

[43] Sueoka T., Koyama K., Hayashi G., Okamoto A. (2018). Chemistry-Driven Epigenetic Investigation of Histone and DNA Modifications. Chem. Rec..

[44] Megee P.C., Morgan B.A., Smith M.M. (1995). Histone H4 and the maintenance of genome integrity. Genes Dev..

[45] Sanders S.L., Portoso M., Mata J., Bahler J., Allshire R.C., Kouzarides T. (2004). Methylation of histone H4 lysine 20 controls recruitment of Crb2 to sites of DNA damage. Cell.

[46] Yan Q., Dutt S., Xu R., Graves K., Juszczynski P., Manis J.P., Shipp M.A. (2009). BBAP monoubiquitylates histone H4 at lysine 91 and selectively modulates the DNA damage response. Mol. Cell..

[47] Yeung K.R., Chiu C.L., Pidsley R., Makris A., Hennessy A., Lind J.M. (2016). DNA methylation profiles in preeclampsia and healthy control placentas. Am. J. Physiol. Heart. Circ. Physiol..

[48] Harmon A.C., Cornelius D.C., Amaral L.M., Faulkner J.L., Cunningham M.W., Wallace K., LaMarca B. (2016). The role of inflammation in the pathology of preeclampsia. Clin. Sci..

[49] Wang Y., Li B., Zhao Y. (2022). Inflammation in Preeclampsia: Genetic Biomarkers, Mechanisms, and Therapeutic Strategies. Front. Immunol..

[50] Chu T., Shaw P., McClain L., Simhan H., Peters D. (2021). High-resolution epigenomic liquid biopsy for noninvasive phenotyping in pregnancy. Prenat. Diagn..

[51] Navajas R., Ramos-Fernandez A., Herraiz I., Galindo A., Bartha J.L., Corrales F., Paradela A. (2022). Quantitative proteomic analysis of serum-purified exosomes identifies putative pre-eclampsia-associated biomarkers. Clin. Proteom..

[52] Than N.G., Romero R., Tarca A.L., Kekesi K.A., Xu Y., Xu Z., Juhasz K., Bhatti G., Leavitt R.J., Gelencser Z. (2018). Integrated Systems Biology Approach Identifies Novel Maternal and Placental Pathways of Preeclampsia. Front. Immunol..

[53] Meyberg R., Boos R., Babajan A., Ertan A.K., Schmidt W. (2000). Intrauterine growth retardation--perinatal mortality and postnatal morbidity in a perinatal center. Z. Geburtshilfe Neonatol..

[54] Duley L. (2009). The global impact of pre-eclampsia and eclampsia. Semin. Perinatol..

